# Identification of a methyltransferase catalyzing the final step of methyl anthranilate synthesis in cultivated strawberry

**DOI:** 10.1186/s12870-017-1088-1

**Published:** 2017-08-31

**Authors:** Jeremy Pillet, Alan H. Chambers, Christopher Barbey, Zhilong Bao, Anne Plotto, Jinhe Bai, Michael Schwieterman, Timothy Johnson, Benjamin Harrison, Vance M. Whitaker, Thomas A. Colquhoun, Kevin M. Folta

**Affiliations:** 1 Horticultural Sciences Department, University of Florida, 1251 Fifield Hall, Gainesville, FL 32611 USA; 2 Tropical Research and Education Center, University of Florida, Homestead, FL USA; 3 Horticultural Research Laboratory, Agriculture Research Service, USDA, Ft. Pierce, FL USA; 4 Environmental Horticultural Sciences Department, University of Florida, Gainesville, FL USA; 5 Gulf Coast Research and Education Center, University of Florida, Wimauma, FL USA; 6 Plant Molecular and Cellular Biology Program, University of Florida, Gainesville, FL USA

**Keywords:** Aroma, Flavor, *Fragaria*, Grape, Methyl anthranilate, Molecular maker, Strawberry, Volatiles

## Abstract

**Background:**

Methyl anthranilate (MA) contributes an attractive fruity note to the complex flavor and aroma of strawberry (*Fragaria* spp.), yet it is rare in modern cultivars. The genetic basis for its biosynthesis has not been elucidated. Understanding the specific genes required for its synthesis could allow  the development of gene/allele-specific molecular markers to speed breeding of flavorful strawberries.

**Results:**

Ripe fruits from individuals in an F1 population resulting from a cross between a MA producer and a non-producer were examined using a bulk-segregant transcriptome approach. MA producer and non-producer transcriptomes were compared, revealing five candidate transcripts that strictly co-segregated with MA production. One candidate encodes an annotated methyltransferase. MA levels are lower when this transcript is suppressed with RNAi, and bacterial cultures expressing the protein produced MA in the presence of anthranilic acid. Frozen fruit powders reconstituted with anthranilic acid and a methyl donor produced MA only if the transcript was detected in the fruit powder. A DNA-based molecular marker was developed that segregates with the MA-producing gene variant.

**Conclusions:**

These analyses indicate that the methyltransferase, now noted *ANTHRANILIC ACID METHYL TRANSFERASE* (*FanAAMT),* mediates the ultimate step of MA production in cultivated strawberry. Identification of this gene and its associated molecular marker may hasten breeding efforts to introduce this important volatile into modern cultivars.

**Electronic supplementary material:**

The online version of this article (doi:10.1186/s12870-017-1088-1) contains supplementary material, which is available to authorized users.

## Background

Methyl anthranilate (MA) is a key volatile of the highly aromatic diploid strawberry, *F. vesca* [1], imparting a key grape note to fruit aroma. MA is rare within commercial octoploid strawberry (*F.* x *ananassa*) germplasm, and has been reported in only a few cultivars, including ‘Mara des Bois’ and ‘Mieze Schindler’ [2]. These octoploid genotypes have been noted for their attractive flavors and aromas, yet they have other shortcomings that limit their cultivation in large-scale production. Combining genetics for enhanced aroma and superior production traits is a long-term strawberry breeding objective, but aroma phenotyping is laborious and time consuming. A molecular understanding of the limiting factors behind MA synthesis should enable marker-driven introgression of this volatile into high-quality commercial strawberries with greater efficiency than phenotypic assessment alone.

Over 360 volatiles have been detected in strawberry [3]. Determining the most important volatile subsets has usually relied on many factors including quantitative abundance [4], odor thresholds [5, 6], or aroma values and human sensory panels [2, 7–9]. These reports do not include MA in their analysis because it is not present in the genotypes tested. Some reports specifically investigated the differences in aroma (including MA) between *F. vesca* and *F.* x *ananassa* [10]. Further work showed that a tetraploid *F. vesca* ‘Rügen’ x *F.* x *ananassa* ‘Valentine’ had MA [11]. MA is also a key volatile used to discriminate between strawberry aroma types [2].

MA is the distinct volatile in the aroma of concord grape (*Vitis labrusca*) [12]. It has been identified in many plants including some apple genotypes [13], citrus blossoms and honey [14–17], as well as in maize where it is released from leaves to deter herbivore feeding [18]. Analysis of the biochemistry of concord grape shows that an anthraniloyl-coenzyme A:methanol acyltransferase is capable of synthesizing MA from acyl coA and methanol [12].

Analysis of the inheritance of MA in strawberry showed that MA was only detectable in 25% of the progeny from a ‘Mieze Schindler’ (MA-positive) x ‘Elsanta’ (MA-negative) cross [19]. This finding further demonstrates the need to develop molecular markers to predict a plant’s potential to produce MA throughout the breeding process. Wide fluctuations in MA abundance have been observed over the growing season [8, 20], so breeding efforts would benefit from molecular markers associated with the capability to produce the compound.

This report analyzes transcriptomes of MA producers and non-producers bulked in silico [21] to identify transcripts correlating with MA accumulation. One candidate correlates well with MA production, and several assays demonstrate that the cognate protein is capable of mediating the ultimate step in MA production.

## Methods

### Plant materials

Two commercial octoploid *Fragaria* x *ananassa* parents were selected for study based on their volatile profiles. University of Florida cultivar ‘Florida Elyana’ (hereafter referred to as ‘Elyana’) is a commercial quality genotype with large fruits and high fruit sugar content and is negative for the “grape-like” volatile methyl anthranilate (MA). ‘Mara des Bois’ has fruit that are relatively small and soft, and the genotype is MA positive. Plants were maintained in the field at the Gulf Coast Research and Education Center (GCREC) in Wimauma, Florida.

Progeny from the ‘Elyana’ x ‘Mara des Bois’ cross were clonally multiplied a temperate summer nursery in 2010. Two runner tips from each of between 130 (+/− 10) seedlings were transplanted into the field at GCREC starting in the 2010–11 growing season. Strawberry fruits were harvested January 20, February 11 and March 18 in 2011, and then January 13, January 31 and March 7 in 2013. The population has been described previously for other volatiles and RNA sequencing analysis [21, 22].

### Strawberry volatile processing

All fruiting progeny were analyzed for volatiles at each harvest except for the RNA-seq combined harvest on December 15, 2011. Five or six fully-ripe fruit (assessed by size and color) per individual were harvested in the morning from the field and stored in a cooler with ice packs until sample processing. Representative ~25 (+/− 3) g samples from each genotype were blended with an equal weight of saturated NaCl (35% NaCl), and the internal volatile standard 3-hexanone was added to a final concentration of 1 ppm. Two 5 ml aliquots from each genotype were aliquoted into 20 ml glass vials and sealed with magnetic crimp caps (Gerstel, Baltimore, MD, USA). Samples were immediately frozen at −80 °C after processing and kept frozen prior to volatile analysis.

### Gas chromatography/mass spectroscopy (GC/MS) analysis

A 2 cm tri-phase SPME fiber (50/30 μm DVB/Carboxen/PDMS, Supelco, Bellefonte, PA, USA) was used to collect and concentrate volatiles prior to analysis on an Agilent 6890 GC coupled with a 5973 N MS detector (Agilent Technologies, Palo Alto, CA, USA). Before analysis, samples were held at 10 °C in a Peltier cooling tray attached to a MPS2 autosampler (Gerstel). All other volatile sampling and analysis methods were as previously described [23]. An authentic MA standard (Sigma Aldrich, St. Louis, MO, USA) was run under the same chromatographic conditions as fruit samples for verification of volatile identity. MA was often found in low abundance and was therefore quantified using a Single Ion Mode method scanning for the major MA ion (ion 119). The peak area of ion 119 was quantified and normalized to the peak area of the internal standard. The normalized peak areas were compared between samples.

### Combined volatile and RNA-seq materials

Volatile and RNA-seq analysis were performed on fruit harvested on December 15, 2011. The collection included fourteen progeny and both parents (‘Elyana’ and ‘Mara des Bois’). Three of these genotypes (‘Mara des Bois’, seedling 098, and seedling 103) were chosen because they were MA positive, and the rest were non-producers. Eight to ten fully-ripe fruit were used from each genetic line. Fruits were cleaned and the calyx was removed. The fruit were split longitudinally, and half of each fruit was processed for volatile analysis and half was frozen in liquid nitrogen and stored at −80 °C for RNA extraction.

### RNA-seq sample RNA extraction

Frozen berries were ground to fine powder in a liquid nitrogen-cooled coffee grinder (KitchenAid Blade Coffee Grinder, St. Joseph, MI). RNA was extracted using a method modified from Chang et al. (2003). Five ml extraction buffer (2% CTAB, 2% PVP (K30), 100 mM Tris, 25 mM EDTA, 2 M NaCl, 0.5 g/L spermidine, 2% β-mercaptoethanol, pH 8.0) at 65 °C was combined with two g fruit powder and vortexed to mix. Samples were incubated at 65 °C for 10 min. Five ml chloroform:isoamyl alcohol (24:1 *v*/v) were added to each sample and homogenized with a polytron at 80% max speed for 1 min. Samples were centrifuged at room temperature (RT) for 10 min at 8000 x g to separate phases. The supernatant was removed to a fresh tube, vortexed with five ml chloroform:isoamyl alcohol (24:1 *v*/v), and centrifuged again as before. The supernatant was transferred to a fresh tube and LiCl was added to a final concentration of 2 M. Samples were then packed in ice and kept at 4 °C overnight. Samples were kept cold, and all transfers were performed with filter tips from this point forward. Samples were centrifuged at 10,000 x g at 4 °C, and for 30 min to pellet RNA. The supernatant was decanted and 500 ul SSTE buffer (1 M NaCl, 0.5% SDS, 10 mM Tris, 1 mM EDTA, pH 8.0) added to resuspend the RNA pellet. The sample was then extracted once with an equal volume of chloroform:isoamyl alcohol (24:1 *v*/v), vortexed, centrifuged at 8000 x g for 10 min at 4 °C, and the supernatant removed to a 2 ml microcentrifuge tube. 2.5 volumes of ice cold 100% ethanol were added to each sample, and RNA was precipitated at −80 °C for 30 min. RNA was then pelleted at 10,000 x g at 4 °C for 30 min. The supernatant was decanted, pellets washed in 500 ul 76% ethanol 0.3 M NaOAC, centrifuged as above, and the supernatant aspirated off. The RNA pellet was dried in a flow hood for 15 min at RT then resuspended in 30 μl 10 mM Tris, 2.5 mM EDTA.

### Transcriptome analysis

Two separate de novo transcriptome assemblies were performed based on ‘Mara des Bois’ and ‘Elyana’ reads. The first one was built using trinity algorithms and was composed by 112,854 contigs. The second assembly was built using CLC genomic workbench algorithms and was composed of 65,894 contigs. Reads that passed quality checks were aligned to the two de novo transcriptome assemblies using CLC genomics workbench (CLC Bio, Denmark). The *F. vesca* genome assembly from Tennessen et al. [24] was used for comparing candidate contigs, and *F. vesca* Genemark Hybrid version 1.1 was used for gene annotations [25].

RNA seq data was generated using protocols for Illumina Genome Analyzer IIx library preparation as previously described [21, 22]. In the present work, all reads were aligned against two de novo transcriptome assemblies derived from reads in the parental lines. One assembly was constructed using the trinity algorithm [26] and one was made using CLC genomics workbench. The reads were sorted and pooled based on MA presence or absence, and then aligned against the assemblies. Several filters were applied in order to identify transcripts present coincident with MA production. The first filter defined a threshold value that defined presence (RPKM >10) and absence (RPKM <5). The second filter identified transcripts that were not detected in at least three genotypes (‘Elyana’ included) but were detected in at least six genotypes. The third filter identified transcripts that were predicted the two different assemblies

### Candidate expression analysis

Quantitative RT-PCR was performed using RNA derived from the same tissues used in the RNA-seq experiment. RNA was isolated with the Norgen Biotek plant/fungi total RNA purification kit (Norgen Biotek, Canada). 150 mg tissue was used per column, and the RNA was DNAse-treated. A cDNA template for qRT-PCR was synthesized using the Impromtu II Reverse Transcriptase kit (Promega, Madison, WI, USA) according to the manufacturer’s instructions. The cDNA was diluted 1:10 prior to qRT-PCR. All qRT-PCR reactions were run in 20 μL reactions using EvaGreen qPCR Mastermix-ROX (Applied Biological Materials Inc., Richmond, BC, Canada). Each reaction contained 10 μL 2× EvaGreen mastermix, 2 μL primer mix (2 μM each), 1 μL 1:10 diluted cDNA, and 7 μL DNase/RNase free water. All qRT PCR primers were designed using qRT primer design tools available online (idtdna.com), and designed to amplify fragments between 95 and 110 base pairs. Each primer-template combination was run with three technical replicates and repeated for at least three biological replicates. A transcript corresponding to a conserved hypothetical protein FaCHP1; [27]) was used as a constitutive reference. The qRT PCR was run on an Applied Biosystems StepOnePlus Real-Time PCR System using StepOne Software (v2.0) (Applied Biosystems, Foster City, CA, USA). The qRT-PCR data was analyzed using the comparative C_T_ method (ΔΔC_T_) following the manufacturer’s direction. A list of qRT-PCR primer sequences is provided in Additional file 1: Table S1.

### Transient expression assay

Transient expression in strawberry fruits by agroinfiltration was performed according to Hoffmann et al. [28]. Briefly, the *Agrobacterium tumefaciens* strain AGL0 [29] containing the pHELLSGATE12 plasmid bearing a head-to-head construct from the 3′ end of the *FanAAMT* transcript and UTR, was grown at 28 °C in Luria–Bertani (LB) medium with appropriate antibiotics. When the culture reached an OD_600_ of 0.8, *Agrobacterium* cells were harvested and resuspended in a modified MacConkey (MMA) medium (Murashige and Skoog salts, 10 mM morpholine ethanesulphonic acid pH 5.6, 20 g · L^−1^ sucrose). The *Agrobacterium* suspension was then injected into multiple sites over the entire fruit while it was still attached to the plant at about 14 d after pollination using a sterile 1 ml hypodermic syringe. The fruits were harvested from 7 to 14 d after injection when ripe and tested for *FanAAMT* transcripts and MA presence. A control plasmid containing a hairpin construct of *FanFAD1* [21] and a comparable injection protocol was used to control for plasmid/infection/injection effects on MA production.

### Volatile extraction from strawberry fruit transient expression assay

Strawberry fruit from transient expression assay were quenched in liquid nitrogen immediately upon harvest and subsequently stored at −80 °C prior to extraction. Frozen berries were crushed and then ground to a fine powder in a liquid nitrogen-cooled coffee grinder (KitchenAid Blade Coffee Grinder, St. Joseph, MI). Volatiles extractions were carried out in technical triplicate for each sample. Volatile compounds were extracted from samples, 250 mg of finely ground powder, using 1 mL of MTBE (Sigma-Aldrich) containing nonyl acetate (Sigma-Aldrich) at 500 ng mL^−1^ as a surrogate standard. Samples were vortexed for 10 s then shaken for 15 min at 1400 RPM at 25 °C in a Thermomixer (Eppendorf). Following 5 min of centrifugation at 4000 x g the MTBE phase was transferred into a glass sample vial. All samples were evaporated under nitrogen gas to a volume of 100 μL for volatile analysis.

### Gas chromatography/mass spectroscopy (GC/MS)

Volatiles were concentrated using a tri-phase SPME fiber (2 cm, 50/30 μm DVB/Carboxen/PDMS, Supelco, Bellefonte, PA, USA) running on an Agilent 6890 GC coupled with a 5973 N MS detector (Agilent Technologies, Palo Alto, CA, USA). Samples were maintained at 4 °C on a Peltier cooling tray attached to a MPS2 autosampler (Gerstel) prior to analysis. Other volatile sampling and analyses were performed as described previously [30]. The volatile 3-hexanone served as an internal control. An authentic methyl anthranilate standard (Sigma Aldrich, St. Louis, MO, USA) was run under the same chromatographic conditions to identify the correct peak.

### MA production in *E. coli*

The full length sequence of *FanAAMT* was cloned in the expression vector pET-28b. The construct was then inserted in the *E.Coli BL21*-CodonPlus (DE3)-*RIPL* strain by heat shock. 5 ml of cultures were grown overnight at 37 °C with shaking. The cultures were then diluted in 50 mL of LB-antibiotics and shaken at room temperature. When OD reached ~0.8–1.0, IPTG was added to 1 mM for transcriptional induction, and various concentrations of anthranilic acid were added to the cultures. 2 mL of cultures were tested for MA production at the induction time (T0) and after 1, 2, 3, 4 and 5 days.

### MA production in fruit protein extracts

Total soluble protein was isolated from 2 g frozen puree from ripe strawberry fruit. A 2:1 ratio of extraction buffer was used, consisting of 100 mM K-phosphate buffer pH 7.8, 30 mg/mL BSA, and 2 mM DTT. To each sample, 0.75 g insoluble PVPP was also added. The supernatant was collected after cold centrifugation. The sample was filtered through a buffer-equilibrated PD-10 desalting column to remove a majority of organic compounds dissolved in the extract. Semi-purified total soluble protein was eluted in 4 mL buffer. For the complete reaction, 1 mL of extract was incubated with 2 mM anthranilate and 2 mM S-Adenosyl methionine (ado-met). Samples were incubated to various time points, together with appropriate negative controls. Prior to headspace sampling with SPME-GC/MS, samples were warmed to 40 °C for 10 min to promote volatilization. Headspace analysis were performed on independent incubations which were sealed immediately following initial mixing and sampled once.

### Molecular marker design

The region around *FanAAMT* was targeted for variations that may be related to a mechanistic explain the presence of the transcript in ‘Mara des Bois’ and absence of detectable transcript in ‘Elyana’. Sequencing around the gene revealed a variation in genomic DNA sequence between ‘Mara des Bois’ and ‘Elyana’. There were conserved differences between the parental alleles. A region with five SNPs in 16 nucleotides was targeted for preferential amplification of a ~1.2 kb product representing ‘Mara des Bois’ and MA producers. A co-amplifying product (~300 bp) is produced in all samples. Primers were forward 5′ GGGATTGAATGCAATTTGTCTATTTTGCCTTTTTTTCTGTA 3′ and reverse 5′ GAACACTAGCATCCCAATCCA 3′.

## Results

### Seasonal MA variation in the ‘Elyana’ x ‘Mara des Bois’ F1 population

**Fig. 1:**
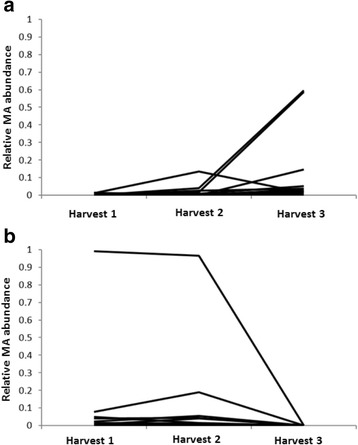
Variability in MA detection over two growing seasons. Each line represents MA detected from strawberries from an individual genotype from the ‘Elyana’ x ‘Mara des Bois’ cross, performed on three separate harvests (January 20, February 11 and March 18 in 2011; January 13, January 31 and March 7 in 2013). **a** MA trends detected for the 2010/11 season including 30 individuals. **b** MA detection in the 2012/13 season including 18 individuals

‘Mara des Bois’, a 1990’s French hybrid known to produce MA, and ‘Elyana’, a Florida cultivar that does not produce MA, were crossed to test MA segregation. Seasonal variability of MA was measured in ‘Elyana’ x ‘Mara des Bois’ fruits, as well as in the progeny at three independent harvests over two growing seasons in uniform fully-expanded, red-ripe stage fruits. Figure 1a depicts MA variability for the general trends observed in 30 individuals over three consecutive harvests. MA production was low with a slight increase in the average MA for all genotypes through the season. Figure 1b shows the MA volatile profiles of 18 individuals propagated and grown during the 2012/13 season. Overall, 37 of 114 progeny tested produced MA, but relative MA production was generally low, and only two genotypes were identified as containing relatively high amounts of MA.

### MA abundance in parents and selected progeny

**Fig. 2:**
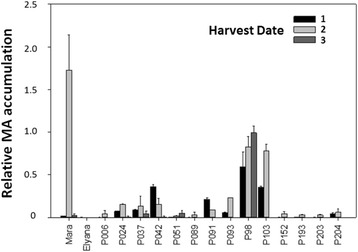
MA detection in the ‘Elyana’ x ‘Mara des Bois’ F1 population over three harvests in 2011/2012. The relative quantity is on the GC/MS peak area of MA, based on two technical replicates as described in Materials and Methods. All data are scaled relative to the internal GC/MS standard 3-hexanone. Harvest dates were January 20, February 11 and March 18 in 2011. *Error bars* represent standard error of the mean

The variability of MA abundance in ripe fruits required repeated sampling in order to obtain an optimal harvest time to conduct RNA-seq experiments. Fig. 2 shows MA detection in the16 genotypes used for volatile analysis and RNAseq over three harvests from three growing seasons. Overall, MA production was detected more often in Harvest 2 compared to Harvests 1 and 3. ‘Mara des Bois’ had the highest level of MA followed by seedlings P098 and P103. Seedlings P024, P037, P042, P091 and P093 showed a relatively low level of MA. MA was not detected in the other individuals. Seedling P098 was the only genotype with relatively high production of MA during all three harvests and seasons.

### Bulk-segregant analysis of transcript pools

Harvest 2 was selected for transcriptome analysis, using the filters described in Materials and Methods. Transcripts not specific to MA producers were eliminated and reduced the candidate list to only five transcripts (Table 1). These candidates were then compared with BLAST against the *Fragaria vesca* reference genome [25] in order to infer putative function from matching sequences. The contigs 7159 and 10,605 matched an unannotated region of the genome. Contigs 12,547 and 5826 aligned to predicted genes (respectively gene03636 and gene32346) but did not represent the full length of the predicted transcripts. Finally, only contig 1885 matched a full length prediction, gene04119. This gene is predicted to encode a jasmonate O-methyltransferase-like protein.

**Table 1:** List of transcripts found exclusively in MA-producing fruits in the ‘Mara des Bois’ x ‘Elyana’ population*. F. vesca* gene annotation is based on the *fvb* assembly

Candidate gene	RPKM value				*F.vesca* reference	Putative annotation	Chromosome position in *F.vesca*
	‘Mara’	‘Elyana’	P098	P103			
contig 1885	131.23	0.34	57.74	85.39	gene04119	jasmonate O-methyltransferase-like	fvb4: 29,995,968..29997295
contig 7159	44.47	0.24	60.64	37.05	-	none	fvb4: 18,630,500..18631978
contig 12,547	26.01	1.75	37.43	33.52	gene03636 (partial)	Uncharacterized membrane protein	fvb4: 26,484,845..26487336
contig 10,605	48.69	0.67	27.46	114.68	-	supported by EST	fvb6: 1,674,840..1675436
contig 5826	26.52	2.61	42.15	41.24	gene32346 (partial)	Transcription factor bHLH120	fvb5: 686,896..688599

### Candidate gene expression during ripening

**Fig. 3:**
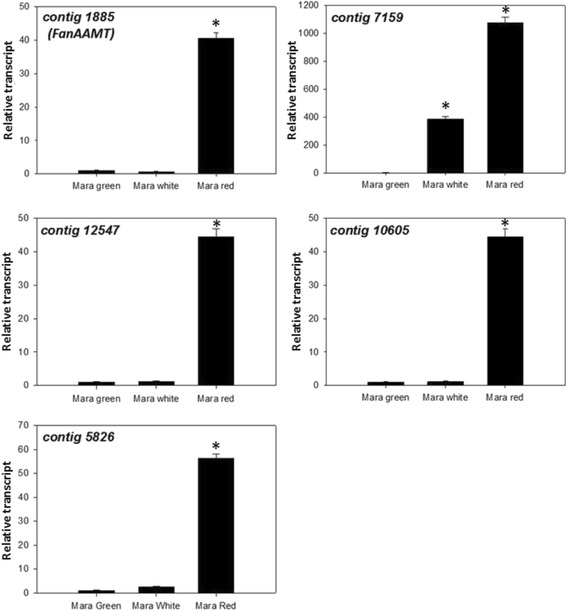
MA candidate gene expression profiles during fruit development. Contig numbers correspond to the De Novo Transcriptome Assembly built based on the ‘Mara des Bois’ transcriptome by using the CLC algorithm. Data from qRT-PCR analysis are shown from one representative experimental  replicate composed of  three technical replicates. *Error bars* represent standard error of the *mean* and *letters* indicate significant differences based on a Tukey’s test, *p* < 0.001

**Fig. 4:**
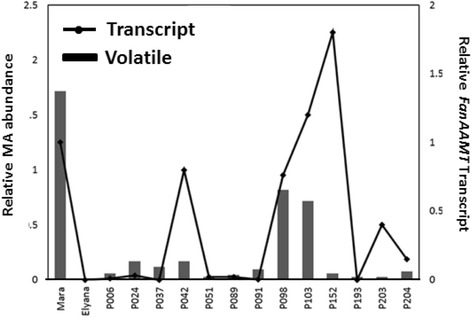
*FanAAMT* transcript accumulation in the MA producing genotypes from the second harvest date. Transcript levels are shown from one representative biological replicate composed of three technical replicates. Error bars represent standard error of the mean

Methyl anthranilate is known to accumulate during ripening, so coincident accumulation of candidate transcripts may infer further function in the process. All candidate transcripts were tested for ripening induction in ‘Mara des Bois’ fruits using qRT-PCR. All candidates displayed a ripening-induced pattern as shown in Fig. 3. Contigs 1885, 12,547, 10,605 and 5826 showed an increase in transcript abundance specifically during the ripe fruit stage and contig 7159 showed progressive increases in transcript abundance from ‘Mara des Bois’ green to white and white to red fruit. No differences were detected in the raw Ct values for the reference transcript *FaCHP1* during ripening, as previously noted [27]. A comparison of the *FanAAMT* transcript level to relative MA level are shown across select progeny (Fig. 4).

**Fig. 5:**
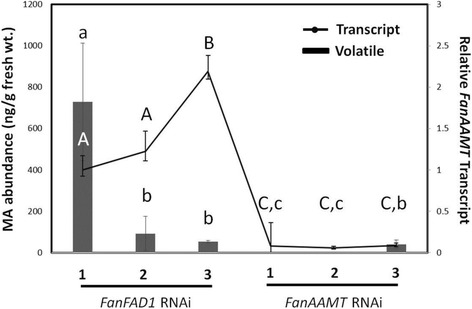
Transient silencing of *FanAAMT* expression in ‘Mara Des Bois’ fruit. A) Absolute abundance of MA in three fruit transiently silenced for *Fatty Acid Desaturase 1* (*FanFAD1*), and *three independent sets of fruits* transiently silenced for *FanAAMT*. B) Relative accumulation of the *FanAAMT* transcript in wild type, *FanFAD1* silenced, and *FanAAMT* silenced fruit. *Error bars* indicate standard error of the *mean*, and *shared letters* indicate no significant differences between lines for the transcript (*capital letters*) and volatile (*small letters*) accumulation based on one-way ANOVA and pairwise *t*-tests

**Fig. 6:**
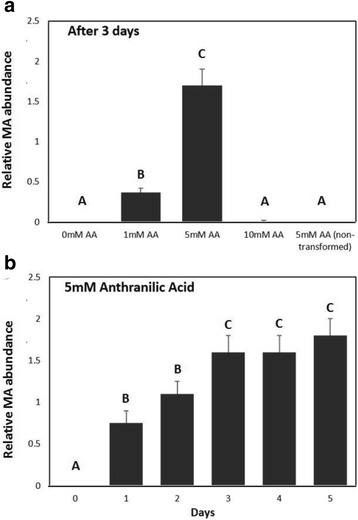
MA production by *E. coli* overexpressing *FanAAMT*. **a** Dose response of MA production after 3 days. Representative data are shown from averaging three independent replicates. **b** Time course of MA production during 5 d with 5 mM of Anthranilic Acid. *Error bars* represent standard error of the *mean*, and *shared letters* are indicative of no significant differences based on a Tukey’s test (*p* < 0.05*)*

**Fig. 7:**
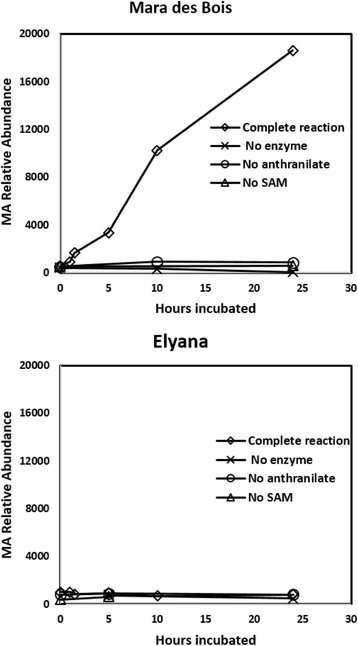
MA production from anthranilic acid in protein extracts from MA positive fruits. Protein extracts were prepared from ‘Mara des Bois’ and ‘Elyana’ fruits. The extracts were incubated with a methyl donor and anthranilic acid. Exclusion of extract, methyl donor or anthranilic acid resulted in no accumulation of MA. Statistical significance (*p* < 0.0001) was established via ANOVA across treatments (F = 110.7) and time (F = 93.9). MA was not detected in extracts from ‘Elyana’

**Fig. 8:**
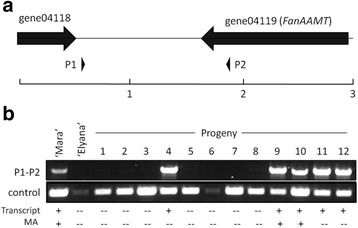
A PCR product derived from *FanAAMT-*adjacent sequence segregates with the potential to produce MA. Panel **a** shows the position of the primers relative to the gene. Panel **b** shows the presence or absence of the associated amplicon in the ‘Mara des Bois’ and ‘Elyana’ parental lines and twelve segregating progeny. A co-amplifying product (shown below) serves as a control for DNA quality, and is reproducibly faint in the ‘Elyana’ parent and some progeny. The same fruits were tested for the presence of the transcript and volatile, demonstrating that the transcript segregates 1:1 with the marker, and is necessary, yet not sufficient, for high-amplitude production of MA

### *FanAAMT* from *Fragaria* × *ananassa*

Because of its putative function, contig 1885 was chosen for further study and renamed *FanAAMT* (*Anthranilic Acid Methyl Transferase*). The *Fragaria* × *ananassa* DNA sequence is 97.12% identical to the *Fragaria vesca* sequence. A SAM-dependent carboxyl methyltransferase domain is predicted from amino acids 15 to 386. To demonstrate relationships with other described methyltransferases and potentially inform function, a neighbor-joining tree was constructed using amino acid sequences of all substrate-characterized, SAM-dependent plant methyl transferases bearing the *Methyl Transferase_7* domain (Additional file 1: Figure S1). The results show that *Fan*AAMT is most similar to a series of strawberry predicted methyltransferases, including one with demonstrated function in jasmonate synthesis [31], followed by jasmonate methyltransferases from *Clarkia brewerii*, Arabidopsis, coffee, and poplar. This sequence is next similar to the characterized AAMTs from maize [18].

### Suppression of *FanAAMT* in fruit agroinfiltration assays

Strawberry fruits may be used in a transient assay to test gene function. Developing fruits are injected with *Agrobacterium* cultures containing a construct of interest, either to suppress or induce target transcript accumulation. Days later, the RNA levels of the relevant transcript may be assayed, along with relevant metabolic queries. Here, ‘Mara des Bois’ fruits were transiently transformed with a hairpin construct for *FanAAMT*. The results show that native transcripts decreased in abundance (Fig. 5). MA was not detected in RNAi suppressed fruits. Fruits treated with an unrelated control construct (*FanFAD1* hairpin) did not show RNAi suppression of the *FanAAMT* expression, and MA was detected. The specificity of the RNAi construct was assessed by measuring the transcript levels of other methyltransferases with similar sequence. The results showed that none of them was strongly affected (Additional file 1: Figure S3). RNA-seq reads representing these other methyltransferase transcripts are also readily detected in non-MA producing fruits (data not shown).

### Evidence of *FanAAMT* activity expressed in *E. coli*

The activity of *FanAAMT* could not be demonstrated after affinity-column purification of the enzyme using a variety of microbial expression systems (not shown). However, methyltransferase activity was detected in crude lysates of *E. coli* producing the *FanAAMT* transcript from a prokaryotic expression vector. MA was detected in headspace only when the cultures were supplied with anthranilic acid (Fig. 6a). A dose-response relationship was observed (Fig. 6a) and showed that after 3 days 5 mM AA cultures produced 5 times more MA than the 1 mM AA cultures. MA was not detected in the 10 mM AA cultures, which also did not grow well. MA was not detected when anthranilic acid was absent from the cultures or when gene expression was not induced with IPTG. A time course was performed with 5 mM anthranilic acid (Fig. 6b). After 3 days the MA accumulation reached a plateau.

### Detection of *FanAAMT* activity in plant protein extracts

It was of interest to examine the potential substrate(s) for MA production. The simple methylation model suggests that anthranilic acid would be methylated by the *FanAAMT* activity to produce MA. This hypothesis is supported by the results in *E. coli*. To answer this question directly, protein extracts were prepared from reconstituted powdered tissue representing MA positive and MA negative lines from the ‘Mara des Bois’ × ‘Elyana’ population. Quantitative RT-PCR was used to test the same tissue for the presence or absence of *FanAAMT* transcripts. Extracts were incubated with anthranilic acid and a methyl donor (ado-Met). Protein extracts from MA producers were capable of producing MA from anthranilic acid and ado-Met, whereas non-producers could not (Fig. 7). MA was readily detected from the protein extracts of high MA producing lines upon incubation with anthranilic acid. Controls lacking enzyme, anthranilic acid, or ado-Met did not produce MA above background levels.

### Molecular marker development

The environmental variability for MA accumulation makes it difficult to screen for truly positive phenotypes, as a negative result in volatile analysis may happen in a genotype capable of producing MA. In this case, DNA-based molecular markers could prove helpful in identifying genotypes containing variants of the enzyme associated with MA production, and further isolate factors responsible for environmental variation. Primers were designed to amplify upstream of, within, and downstream of gene *FanAAMT*. One primer set amplified a product of ~350 bp in all lines within the population. A second product of ~1.5 kb was amplified in all genotypes producing MA (Fig. 8). The product also was amplified in some non-producers. The same primer pair did not amplify the 1.5 kb band corresponding to MA production in other populations tested (data not shown).

## Discussion

Methyl anthranilate (MA) is an important volatile in many plants including strawberry [10, 12] and some apples [13], and is released form the maize leaves in response to herbivory [18]. It is absent from the aromas of most wine grapes (*Vitis vinifera*), but is recognized as a distinctive and desirable “foxy” note in Concord grapes (*Vitis labrusca*) [12]. Analyses vary, but some trials have found an association between MA and favorable consumer ratings of “fruity” and “aromatic” [32]. MA is a conspicuous note in the aroma of the diploid strawberry, but is very rare in commercial octoploid germplasm [9]. In order to enhance its presence in modern fruit varieties, it is of interest to first identify the genes required for its synthesis because of phenotyping challenges. Variation in structure and/or gene expression may describe the biology underlying the trait, as well as provide a basis for molecular marker development. This report implements bulk transcriptome analysis of progeny segregating for MA production, and identifies a set of transcripts that correlate with its presence. One of them is shown to encode a protein capable of catalyzing the ultimate step in MA synthesis.

Discovery of genes required for MA production has been approached using a variety of genetic methods, but analyses indicate at least two genes are required for its accumulation. Their identification is complicated by the fact that MA production is highly dependent on environmental conditions, as shown in Fig. 1. MA was detected in 24% (46 out of 189) of the progeny between ‘Frau Mieze Schindler’ (MA donor) and ‘Elsanta’ [33]. These results match well with the present study where MA was detected in 32% (37 out of 114) of progeny between ‘Elyana’ and ‘Mara des Bois’. The slight difference may be attributed to the small population size and/or environmental fluctuations that make it challenging to identify true MA-positive genotypes. For single locus control under disomic inheritance (segregating in a single subgenome of octoploid strawberry) and in a cross of a producer by a non-producer, 50% producers would be expected in the progeny. Negative effects of minor loci or decreased penetrance of the trait due to environmental effects could account for the 24–32% of producing progeny observed. Alternatively, a two-gene model in which dominant alleles at two separate and unlinked loci are required for MA production in a cross of a producer by a non-producer could result in 25% producers in the progeny.

The diploid strawberry is estimated to contain ~34,000 genes [25]. The number of allelic variants in the octoploid is assumed to be larger, especially because of the multiple subgenomes present. A bulk-segregant transcript analysis was performed, computationally pooling the clear and consistent producers with the ‘Mara des Bois’ parent, and comparing that data set against one assembled from non-producers. Only five transcripts were detected to be common to the MA producers. These candidates are listed in Table 1. All five are ripening induced (Fig. 3), and correlate temporally with MA accumulation. Each of the candidates is consistently abundant in the ‘Mara des Bois’ (MA donor) parent and not detectable above thresholds in the MA-negative parent ‘Elyana’.

One transcript was annotated as “jasmonate-methyl-transferase-like”. It shares most similarity with a demonstrated jasmonic acid carboxyl methyltransferase that is expressed in strawberry fruits, and the transcript increases in abundance during ripening [31]. The cognate protein contained a predicted *Methyltransferase 7* domain. The transcript represents one of thirteen methyltransferase proteins in the diploid strawberry genome (shown in Additional file 1: Figure S1). This class of proteins has been functionally characterized in several other species. The *FanAAMT* transcript encodes a protein most similar to the characterized *Zea mays* AAMT1, AAMT2, and AAMT3 benzenoid carboxyl methyltransferases, which have a preferred substrate of anthranillic acid [18]. Because of its similarity to the maize enzyme, the corresponding gene was annotated as *FanAAMT,* and subsequent assays would demonstrate that anthranillic acid could indeed act as a substrate for this enzyme as well (Figs. 6 and 7). The transcript also correlates well with the accumulation of MA at other harvest points and in other genetic backgrounds, such as the older variety ‘Frau Mieze Schindler’ (Additional file 1: Fig. S2).

The *FanAAMT* transcript was detected in some non-producers, consistent with the two-gene model proposed for MA production [1]. MA was also detected in some fruits where the transcript was detected at low levels, suggesting that this enzyme may be present and active in fruits at some basal level, but then increases in abundance and/or activity under permissible developmental and/or environmental conditions. Similar to maize, the enzyme was hypothesized to catalyze methylation of anthranilic acid to produce methyl anthranilate and is necessary, but not sufficient, for its production. This hypothesis was tested in several ways. Agroinfiltration of an RNAi construct for *FanAAMT* suppressed endogenous transcript levels in ‘Mara des Bois’ fruits, and the same fruits did not accumulate MA (Fig. 5). Control fruits treated with an unrelated RNAi construct maintained detectable *FanAAMT* transcript accumulation as well as MA production.

Efforts to affinity purify a functional tagged enzyme were not successful. However, *E.coli* cultures containing the *FanAAMT* construct produced MA in the presence of anthranilic acid (Fig. 7). The cultures did not produce MA without the *FanAAMT* sequence or without the anthranilic acid substrate. Higher concentrations of MA inhibit the growth of the culture, consistent with previous observations﻿ of antimicrobial activity. Taken together these data indicate that *FanAAMT* catalyzes the formation of methyl anthranilate by methylation of anthranilic acid. Additional tests were performed on crude protein extracts from strawberry fruits shown to contain (or not contain) the *FanAAMT* transcript. In the presence of anthranilic acid and a methyl donor (ado-met), the extracts from *FanAAMT*-transcript containing genotypes were capable of producing MA. These trials also demonstrated that anthranilic acid is a suitable substrate for the *FanAAMT* enzyme produced in vitro as well as in vivo.

These results indicate that the method of production in strawberry is different from that observed in some other fruits. There are at least two ways to produce carboxyl methyl esters of anthranilic acid [18]. In Concord grapes (*Vitis labrusca*) MA is catalyzed by an anthranilate CoA ligase activity that first carries out an ATP dependent reaction between anthranoyl-CoA and anthranilic acid, followed by an alcohol acyl transferase activity with methanol to form the grape aroma [12]. The alcohol acyl-transferase activity is abundant in Concord grapes (~0.5% of total protein) and can accept a series of substrates and alcohols to produce an array of esters. On the other hand the mechanism in strawberry is identical to that of SABATH methyltransferases, which have been shown to methylate anthranilic acid in the presence of Ado-Met both in vitro and in vivo [18, 34].

Anthranilic acid represents a divergence of primary and secondary metabolism. It is derived from chorismic acid  and then is converted to tryptophan. This position at a branch point in metabolism may explain its environmental variability. Its conversion to a secondary metabolite like methyl anthranilate may be permitted as less substrate is required for amino acid synthesis, or perhaps even auxin production. MA accumulation occurs as receptacle expansion ends. Receptacle expansion is auxin-driven. The inverse relationship between MA accumulation and a decline in auxin-driven processes is consistent with the hypothesis that both the aroma compound and hormone are derived from a common substrate, and the abundance of the aroma is dependent on the decreased demand for auxin synthesis.

The results are significant because understanding the gene necessary for the ultimate step in MA synthesis sets a foundation for marker development in cultivated strawberry. The rare occurrence of MA in octoploid germplasm and its environmental variability make breeding for MA quite challenging. Molecular markers corresponding to the causal genes or alleles would be tremendously beneficial to identify plants containing at least of some the fundamental genetic components required for MA production. A sequence variation occurring 3′ of the *FanAAMT* gene can be detected using PCR, producing an amplicon only in plants capable of producing MA (Fig. 8). Using the primer pair shown in Panel A, a ~ 1.5 kbp product may be amplified in plants capable of MA production (Panel B), including the ‘Frau Meize Schindler’ (MA positive) variety (not shown). The sequence variation correlates exactly with the ability to detect the transcript, yet not always with the volatile, indicating that the detected variant is necessary, but not sufficient, to identify an MA producing genotype. A co-amplifying band served as a control, and was reproducibly amplified at a low level in ‘Elyana’ and some segregating varieties. Additional cursory tests in other MA-segregating populations did not follow the same pattern, indicating that the utility of this specific molecular marker may be limited (C. Barbey and K. Folta, unpublished).

Identification of the transcript, protein, substrate, and molecular marker corresponding to the ultimate step in MA synthesis are first steps toward breeding more precisely for this fruity volatile. Understanding this mechanism also allows more focused efforts on other genes that may contribute to MA accumulation and its ranging environmental variability. These processes may possibly be governed by any of the other four transcripts identified. Ultimately these trials enhance our understanding of the molecular basis of fruity and floral volatiles so that relevant genes can be introduced into common selections, producing better flavored fruits. Such products would find favor with consumers, supporting healthful eating habits and increasing profitability for strawberry growers.

## Conclusion

Consumers agree that there is room for improvement in strawberry flavor. The identification of genes that strongly contribute to fruity-floral volatile production and the development of corresponding gene-based molecular markers are logical steps in hastening breeding for improved sensory traits. In this report a gene encoding a methyltransferase (*FanAAMT*) was identified using bulk-segregant transcriptome analysis, and a molecular marker was devised that can identify genotypes with a higher likelihood of producing the grape aroma volatile. It is clear that *FanAAMT* is likely only modulating the amplitude of MA accumulation, suggesting that additional genes are required for basal MA production. Those genes will be the subject of future studies in the pursuit of better tasting berries that will benefit growers and consumers alike.

## Additional file

Additional file 1:
**S1.** Neighbor-joining tree demonstrating relatedness among proteins containing the methyltransferase_7 domain in strawberry, as related to similar proteins with demonstrated function in other plants. **S2.** Correlation of the *FanAAMT* transcript and MA detection in other producers. **S3.** Demonstration of RNAi effects on non-target transcripts with similar domain sequences. (DOCX 310 kb)

## Abbreviations

*Fan*: *Fragaria x ananassa*
*Fvb*: *Fragaria vesca ssp. bracteata*
*Fve*: *Fragaria vesca*
MA: methyl anthranilate
