# Supplementary material for: Identification of a methyltransferase catalyzing the final step of methyl anthranilate synthesis in cultivated strawberry
Source: BMC Plant Biol. 2017 Aug 31;17:147. doi: 10.1186/s12870-017-1088-1 (PMC5580298; doi:10.1186/s12870-017-1088-1)
Supplement: Additional file 1: — S1. Neighbor-joining tree demonstrating relatedness among proteins containing the methyltransferase_7 domain in strawberry, as related to similar proteins with demonstrated function in other plants. S2. Correlation of the FanAAMT transcript and MA detection in other producers. S3. Demonstration of RNAi effects on non-target transcripts with similar domain sequences. (DOCX 310 kb) [file 12870_2017_1088_MOESM1_ESM.docx]

Figure S1.

Neighbor-joining tree depicting the relationships between FanAAMT1 and other substrate-characterized, SAM-dependent, proteins containing the methyl transferase (MethylTrans_7 ) domain across plant species. Substrates are noted here: JMT- Jasmonate, SAMT – Salicylic acid, BSMT – Benzoic Acid/Saicylic acid, AAMT – Anthranilic Acid, CCMT – Cinnamate/4-coumarate, GAMT – Gibberellic acid, FAMT – Farnasylic Acid. The genes encoding them include: *CbSAMT, Clarkia breweri* SAMT (AF133053); *AmSAMT, Antirrhinum majus* SAMT (AF515284); *SfSAMT, Stephanotis floribunda* SAMT (AJ308570); *HcSAMT, Hoya carnosa* SAMT (AJ863118); *DwSAMT, Datura wrightii* SAMT (EF472972); *AmBAMT, Antirrhinum majus* BAMT (AF198492); *NsBSMT, Nicotiana suaveolens* BSMT (AJ628349); *AtBSMT, Arabidopsis thaliana* BSMT (BT022049); *AlBSMT, A. lyrata* BSMT (AY224596); *AbSAMT, Atropa belladonna* SAMT (AB049752); *OsBSMT1, Oryza sativa* BSMT1 (XM467504); *PhBSMT, Petunia hybrida* BSMT (AY233465); *OsIAMT1, O. sativa* IAMT1 (EU375746); *PtIAMT1, Populus trichocarpa* IAMT1(XP_002298843); *AtIAMT, A. thaliana* IAMT (AK175586); *AtGAMT1, A. thaliana* GAMT1 (At4 g26420); *AtGAMT2, A. thaliana* GAMT2 (At5 g56300); *CaCaS1, Coffea arabica* caffeine synthase 1 (AB086414); *CaXMT1, C. arabica* XMT1 (AB048793); *CaDXMT1, C. arabica* DXMT1 (AB084125)

Figure S2. Correlation between relative transcript levels and the presence of MA in three different genotypes on a separate harvest date. Error bars represent standard error of the mean.

Figure S3. Relative transcript accumulation of several carboxy methyltransferase family genes in *FanAAMT* silenced fruit. *FanFAD1* silenced fruits and non-treated fruits represent negative controls.

Table S1. qPCR primers used for determining transcription of other carboxyl methyltransferase family genes in agroinfiltrated ‘Mara des Bois’ fruit.

Figure S1


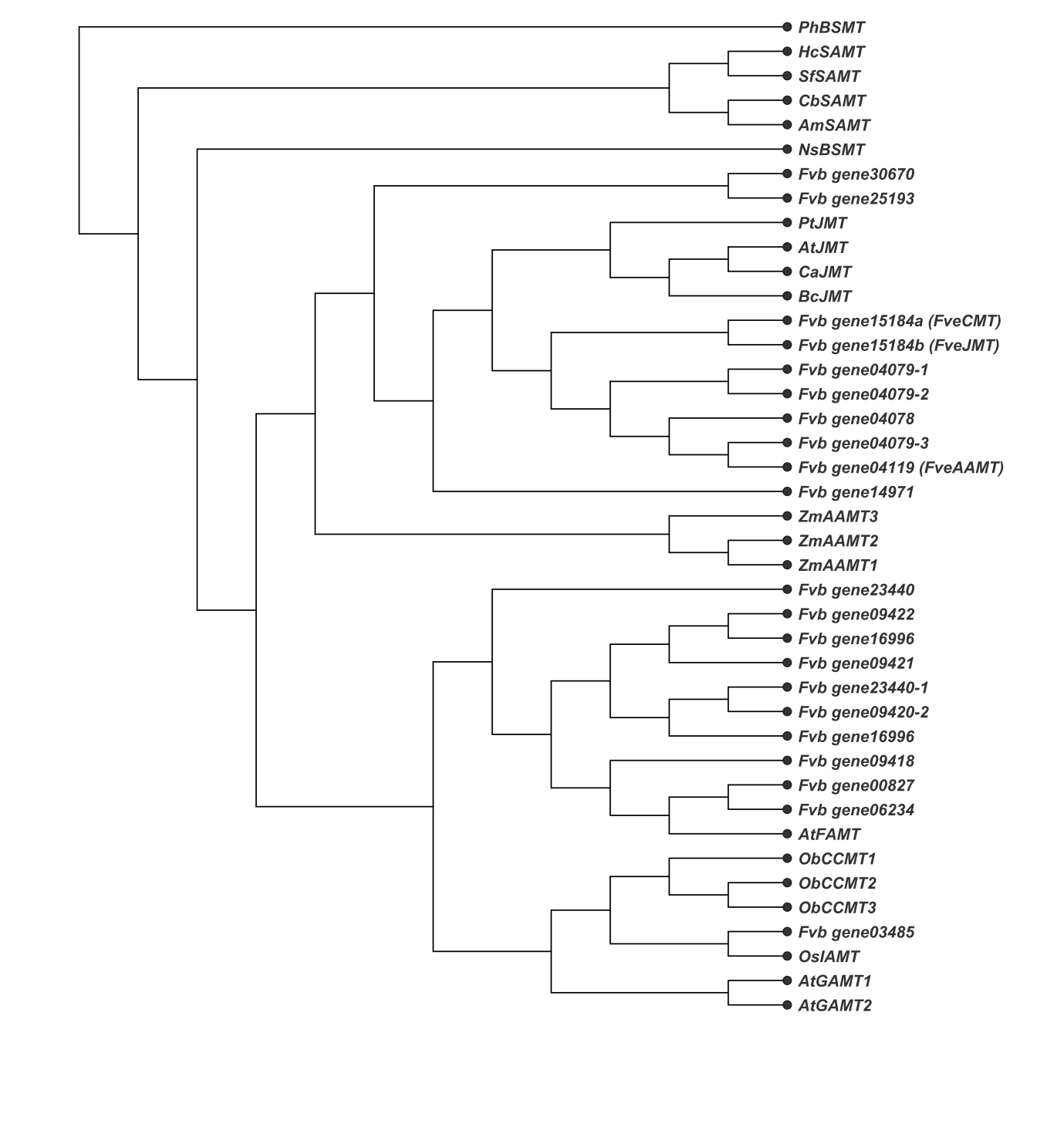


Figure S2


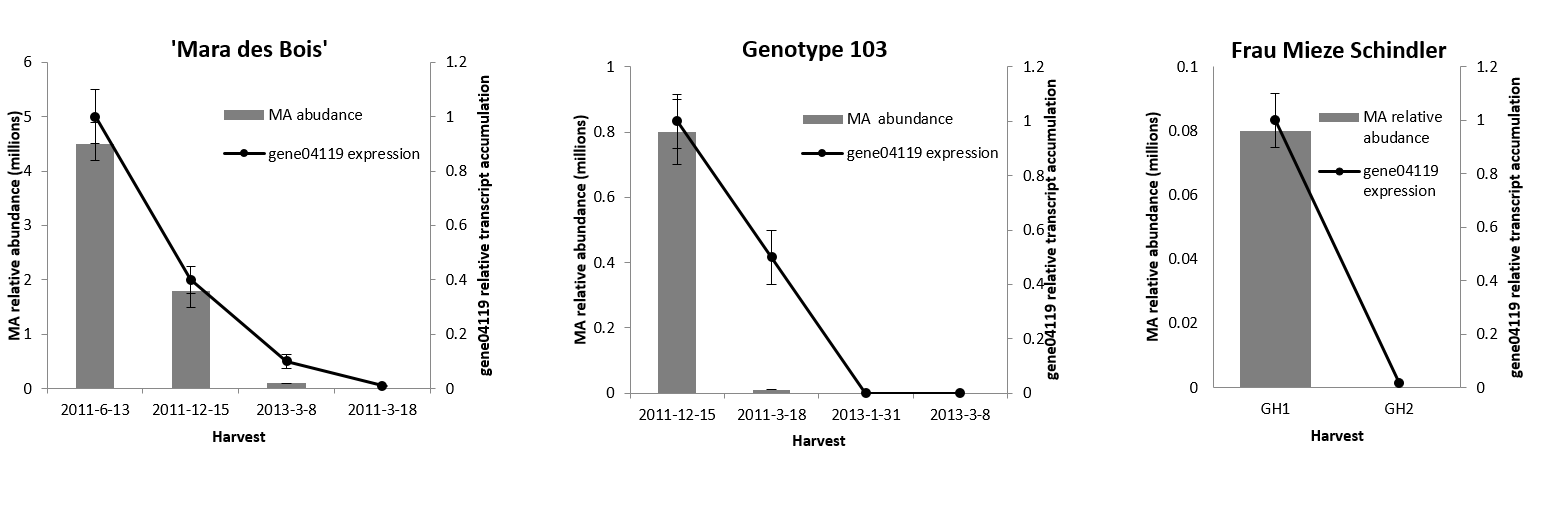


Figure S3


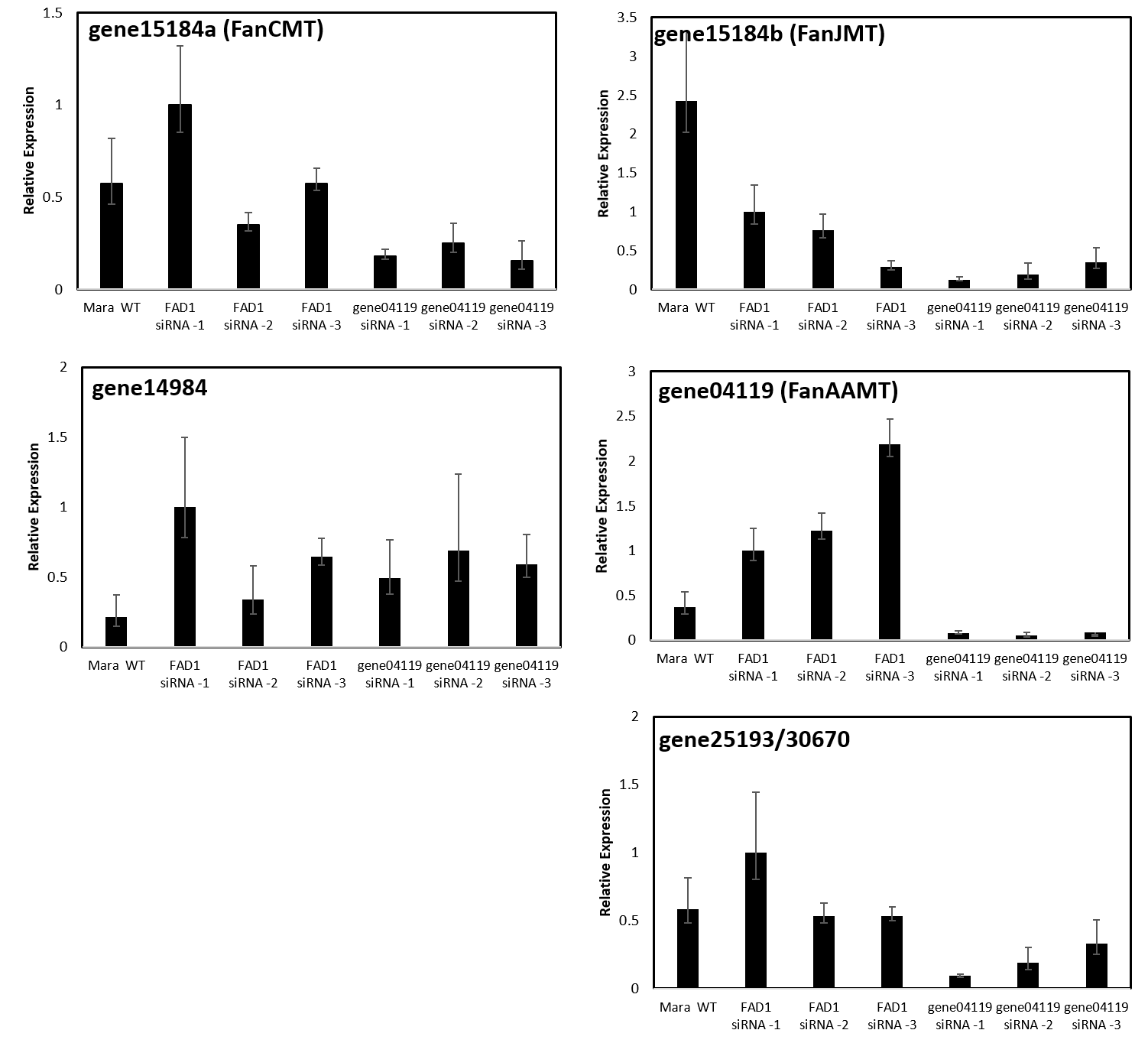


Table S1

| gene04119 | Fwd | CCATGTGCAGGTGAGTTGATAA |
| --- | --- | --- |
| gene04119 | Rev | CAGGAACACTAGCATCCCAATC |
| gene25193/30670 | Fwd | GACCATGGGCAGCATAAA |
| gene25193/30670 | Rev | CTTGTCCTCCTCGATCAAAC |
| gene15184b | Fwd | GACCAAACTCCTTGCTACTC |
| gene15184b | Rev | CAGTGTTGATGACTGGAACT |
| gene15184a | Fwd | GGAGGAGGAAGAATGGTATTG |
| gene15184a | Rev | TCATCAATGCAAGGGCTAATA |
| gene14984 | Fwd | TTTCCAAGGGAGAAGTTGAG |
| gene14984 | Rev | CTCACTGCATCTTCTAACTCAT |
| contig21335 | Fwd | TGCATATATCAAGCAACTTTACACTGA |
| contig21335 | Rev | ATAGCTGAGATGGATCTTCCTGTGA |

**References**

**Aitken KS, Jackson PA, McIntyre CL** (2005) A combination of AFLP and SSR markers provides extensive map coverage and identification of homo(eo)logous linkage groups in a sugarcane cultivar. Theor Appl Genet **110:** 789-801

**Amaya I, Pillet J, Folta KM** (2016) Identification of Genes Responsible for Natural Variation in Volatile Content Using Next-Generation Sequencing Technology. Plant Signal Transduction: Methods and Protocols**:** 37-45

**Attaway JA, Pieringer AP, Barabas LJ** (1966) The origin of citrus flavor components-I: The analysis of citrus leaf oils using gas-liquid chromatography, thin-layer chromatography, and mass spectrometry. Phytochemistry **5:** 141-151

**Cardello AV** (1995) Food Quality - Relativity, Context and Consumer Expectations. Food Quality and Preference **6:** 163-170

**Carrasco B, Hancock JF, Beaudry RM, Retamales JB** (2005) Chemical composition and inheritance patterns of aroma in Fragaria× ananassa and Fragaria virginiana progenies. HortScience **40:** 1649-1650

**Chambers A, Pillet J, Plotto A, Bae J, Whitaker V, Folta K** (2014) Identification of a Strawberry Flavor Gene Candidate Using an Integrated Genetic-Genomic-Analytical Chemistry Approach. BMC Genomics **15:** 217

**Chambers A, Whitaker VM, Gibbs B, Plotto A, Folta KM** (2012) Detection of the linalool-producing NES1 variant across diverse strawberry (Fragaria spp.) accessions. Plant Breeding **131:** 437-443

**Clancy M, Rosli H, Chamala S, Barbazuk W, Civello P, Folta K** (2013) Validation of reference transcripts in strawberry (Fragaria spp.). Molecular Genetics and Genomics **288:** 671-681

**Flamini G, Cioni PL, Morelli I** (2003) Use of solid-phase micro-extraction as a sampling technique in the determination of volatiles emitted by flowers, isolated flower parts and pollen. Journal of Chromatography a **998:** 229-233

**Hirvi T, Honkanen E** (1982) The volatiles of two new strawberry cultivars,“Annelie” and „Alaska Pioneer”, obtained by backcrossing of cultivated strawberries with wild strawberries, fragaria vesca, rügen and fragaria virginiana. Zeitschrift für Lebensmittel-Untersuchung und Forschung **175:** 113-116

**Hoffmann T, Kalinowski G, Schwab W** (2006) RNAi-induced silencing of gene expression in strawberry fruit (Fragaria x ananassa) by agroinfiltration: a rapid assay for gene function analysis. Plant J **48:** 818-826

**Jabalpurwala FA, Smoot JM, Rouseff RL** (2009) A comparison of citrus blossom volatiles. Phytochemistry **70:** 1428-1434

**Krokida MK, Philippopoulos C** (2006) Volatility of apples during air and freeze drying. Journal of Food Engineering **73:** 135-141

**Köllner TG, Lenk C, Zhao N, Seidl-Adams I, Gershenzon J, Chen F, Degenhardt J** (2010) Herbivore-Induced SABATH Methyltransferases of Maize That Methylate Anthranilic Acid Using S-Adenosyl-l-Methionine. Plant Physiology **153:** 1795-1807

**Larsen M, Poll L** (1992) Odour thresholds of some important aroma compounds in strawberries. Z. Lebensum. Unters. Forsch. **195:** 120-123

**Larsen M, Poll L, Olsen CE** (1992) Evaluation of the aroma composition of some strawberry (Fragaria ananassa Duch) cultivars by use of odour threshold values. Z Lebensm Unters Forsch **195:** 536-539

**Lazo GR, Stein PA, Ludwig RA** (1991) A DNA transformation-competent Arabidopsis genomic library in Agrobacterium. Biotechnology (N Y) **9:** 963-967

**Maarse H** (1991) Volatile Compounds in Foods and Beverages. Marcel Dekker, New York

**Nozal MaJ, Bernal JL, Toribio L, Jiménez JJ, Martı́n MaT** (2001) High-performance liquid chromatographic determination of methyl anthranilate, hydroxymethylfurfural and related compounds in honey. Journal of Chromatography A **917:** 95-103

**Olbricht K, Grafe C, Weiss K, Ulrich D** (2008) Inheritance of aroma compounds in a model population of Fragaria × ananassa Duch. Plant Breeding **127:** 87-93

**Olbricht K, Grafe C, Weiss K, Ulrich D** (2008) Inheritance of aroma compounds in a model population of Fragaria× ananassa Duch. Plant Breeding **127:** 87-93

**Olbricht K, Ulrich D, Weiss K, Grafe C** (2011) Variation in the Amounts of Selected Volatiles in a Model Population of Fragaria x ananassa Duch. As Influenced by Harvest Year. Journal of Agricultural and Food Chemistry **59:** 944-952

**Preuß A, Augustin C, Figueroa CR, Hoffmann T, Valpuesta V, Sevilla JF, Schwab W** (2014) Expression of a functional jasmonic acid carboxyl methyltransferase is negatively correlated with strawberry fruit development. Journal of Plant Physiology **171:** 1315-1324

**Pyysalo T, Honkanen E, Hirvi T** (1979) Volatiles of Wild Strawberries, Fragaria-Vesca L, Compared to Those of Cultivated Berries, Fragaria X Ananassa Cv Senga Sengana. Journal of Agricultural and Food Chemistry **27:** 19-22

**Schieberle P, Hofmann T** (1997) Evaluation of the character impact odorants in fresh strawberry juice by quantitative measurements and sensory studies on model mixtures. Journal of Agricultural and Food Chemistry **45:** 227-232

**Schreier P** (1980) Quantitative composition of volatile constituents in cultivated strawberries, Fragaria ananassa cv. Senga Sengana, Senga Litessa and Senga Gourmella. Journal of the Science of Food and Agriculture **31:** 487-494

**Schulz MH, Zerbino DR, Vingron M, Birney E** (2012) Oases: robust de novo RNA-seq assembly across the dynamic range of expression levels. Bioinformatics **28:** 1086-1092

**Shulaev V, Sargent DJ, Crowhurst RN, Mockler TC, Folkerts O, Delcher AL, Jaiswal P, Mockaitis K, Liston A, Mane SP** (2011) The genome of woodland strawberry (Fragaria vesca). Nat Genet **43:** 109-116

**Shulaev V, Sargent DJ, Crowhurst RN, Mockler TC, Folkerts O, Delcher AL, Jaiswal P, Mockaitis K, Liston A, Mane SP, Burns P, Davis TM, Slovin JP, Bassil N, Hellens RP, Evans C, Harkins T, Kodira C, Desany B, Crasta OR, Jensen RV, Allan AC, Michael TP, Setubal JC, Celton JM, Rees DJ, Williams KP, Holt SH, Rojas JJ, Chatterjee M, Liu B, Silva H, Meisel L, Adato A, Filichkin SA, Troggio M, Viola R, Ashman TL, Wang H, Dharmawardhana P, Elser J, Raja R, Priest HD, Bryant DW, Fox SE, Givan SA, Wilhelm LJ, Naithani S, Christoffels A, Salama DY, Carter J, Girona EL, Zdepski A, Wang W, Kerstetter RA, Schwab W, Korban SS, Davik J, Monfort A, Denoyes-Rothan B, Arus P, Mittler R, Flinn B, Aharoni A, Bennetzen JL, Salzberg SL, Dickerman AW, Velasco R, Borodovsky M, Veilleux RE, Folta KM** (2011) The genome of woodland strawberry (Fragaria vesca). Nat Genet**:** 109-116

**Tennessen JA, Govindarajulu R, Ashman T-L, Liston A** (2014) Evolutionary origins and dynamics of octoploid strawberry subgenomes revealed by dense targeted capture linkage maps. Genome biology and evolution **6:** 3295-3313

**Ulrich D, Hoberg E, Olbricht K** (2006) Flavour control in strawberry breeding by sensory and instrumental methods. Acta Horticulturae **708:** 579-584

**Ulrich D, Hoberg E, Rapp A, Kecke S** (1997) Analysis of strawberry flavour - discrimination of aroma types by quantification of volatile compounds. Zeitschrift Fur Lebensmittel-Untersuchung Und-Forschung a-Food Research and Technology **205:** 218-223

**Ulrich D, Komes D, Olbricht K, Hoberg E** (2007) Diversity of aroma patterns in wild and cultivated Fragaria accessions. Genetic Resources and Crop Evolution **54:** 1185-1196

**Wang J, De Luca V** (2005) The biosynthesis and regulation of biosynthesis of Concord grape fruit esters, including 'foxy' methylanthranilate. Plant J **44:** 606-619

**Zhao N, Guan J, Ferrer J-L, Engle N, Chern M, Ronald P, Tschaplinski TJ, Chen F** (2010) Biosynthesis and emission of insect-induced methyl salicylate and methyl benzoate from rice. Plant Physiology and Biochemistry **48:** 279-287
